# Bioenergetic Requirements and Spatiotemporal Profile of Nerve Growth Factor Induced PI3K-Akt Signaling Along Sensory Axons

**DOI:** 10.3389/fnmol.2021.726331

**Published:** 2021-09-24

**Authors:** Rajiv Sainath, Gianluca Gallo

**Affiliations:** ^1^Shriners Hospitals Pediatric Research Center, Lewis Katz School of Medicine, Temple University, Philadelphia, PA, United States; ^2^Department of Neural Sciences, Lewis Katz School of Medicine, Temple University, Philadelphia, PA, United States

**Keywords:** axon, PI3K, Akt, mitochondria, glycolysis

## Abstract

Nerve Growth Factor (NGF) promotes the elaboration of axonal filopodia and branches through PI3K-Akt. NGF activates the TrkA receptor resulting in an initial transient high amplitude burst of PI3K-Akt signaling followed by a maintained lower steady state, hereafter referred to as initiation and steady state phases. Akt initially undergoes phosphorylation at T308 followed by phosphorylation at S473, resulting in maximal kinase activation. We report that during the initiation phase the localization of PI3K signaling, reported by visualizing sites of PIP3 formation, and Akt signaling, reflected by Akt phosphorylation at T308, correlates with the positioning of axonal mitochondria. Mitochondrial oxidative phosphorylation but not glycolysis is required for Akt phosphorylation at T308. In contrast, the phosphorylation of Akt at S473 is not spatially associated with mitochondria and is dependent on both oxidative phosphorylation and glycolysis. Under NGF steady state conditions, maintenance of phosphorylation at T308 shows dual dependence on oxidative phosphorylation and glycolysis. Phosphorylation at S473 is more dependent on glycolysis but also requires oxidative phosphorylation for maintenance over longer time periods. The data indicate that NGF induced PI3K-Akt signaling along axons is preferentially initiated at sites containing mitochondria, in a manner dependent on oxidative phosphorylation. Steady state signaling is discussed in the context of combined contributions by mitochondria and the possibility of glycolysis occurring in association with endocytosed signalosomes.

## Introduction

Nerve growth factor (NGF) regulates multiple aspects of the development of sensory neurons including morphology. NGF rapidly promotes the elaboration of the growth cone and induces the formation of axon collateral branches through the promotion of actin filament polymerization and microtubule tip dynamics (Gallo and Letourneau, 2000; Niewiadomska et al., 2011; Pacheco and Gallo, 2016; Armijo-Weingart and Gallo, 2017). NGF elicits its morphogenetic effects through the activation of the TrkA receptor that in turn activates multiple downstream signaling pathways (Huang and Reichardt, 2003; Reichardt, 2006). The PI3K-Akt signaling axis, downstream of TrkA, mediates the NGF-induced formation of axon collateral branches through multiple cellular effects required for the initiation of axon branching. PI3K-Akt signaling is required for the promotion of axonal actin filament dynamics underlying the emergence of axonal filopodia, the activation of intra-axonal protein synthesis of actin regulatory proteins and the fission of axonal mitochondria (Ketschek and Gallo, 2010; Spillane et al., 2012, 2013; Armijo-Weingart et al., 2019). PI3K signaling also mediates the effects of NGF on microtubule growth and the hyperpolarization of mitochondria (Zhou et al., 2004; Verburg and Hollenbeck, 2008). The positioning of axonal mitochondria along axons determines sites where axon branches can form (Courchet et al., 2013; Spillane et al., 2013; Tao et al., 2014; Sainath et al., 2017a; Wong et al., 2017; Lewis et al., 2018; Smith and Gallo, 2018; Rangaraju et al., 2019) and in the context of NGF-induced branching also sites of preferential intra-axonal protein synthesis of required actin regulatory proteins (Spillane et al., 2013).

ATP is generated through two main sources; oxidative phosphorylation in the mitochondrion and glycolysis. Mitochondrial oxidative phosphorylation has been shown to be a required component of both regenerative and developmental axon extension (Patrón and Zinsmaier, 2016; Zhou et al., 2016; Sainath et al., 2017b; Sheng, 2017; Smith and Gallo, 2018). The targeting of mitochondria to growth cones promotes regeneration and the evacuation of mitochondria from growth cones is promoted by axon inhibitory signals (Morris and Hollenbeck, 1993; Han et al., 2016; Sainath et al., 2017b). Signals that promote axon extension and branching increase mitochondrial function and those that inhibit branching impair mitochondrial function (Verburg and Hollenbeck, 2008; Pacelli et al., 2015; Sainath et al., 2017a). Mitochondrial oxidative phosphorylation also regulates presynaptic transmission, at least in subsets of synapses wherein mitochondria are targeted. The role of glycolysis in axonal biology has received less attention than oxidative phosphorylation but it has been shown to regulate aspects of synaptic physiology (Ashrafi and Ryan, 2017). In developing central and peripheral nervous system neurons the contribution of glycolysis to net ATP levels is greatest during early embryogenesis and then wanes as oxidative phosphorylation becomes a more prominent source (reviewed in Gallo, 2020). Glycolytic enzymes are found throughout the axons of developing sensory neurons and glycolysis contributes to sensory axon extension and growth cone dynamics (Ketschek et al., 2021). Glycolytic enzymes associate with fast transport vesicles and “on board” glycolysis powers the transport of the vesicles while mitochondria transport depends on oxidative phosphorylation independent of glycolysis (Spillane et al., 2013; Zala et al., 2013; Hinckelmann et al., 2016). The contributions of mitochondrial oxidative phosphorylation and glycolysis in signaling by extracellular factors have not been addressed.

NGF-TrkA signaling involves extensive phosphorylation, endocytosis of receptors and transport of the ensuing signaling endosomes (signalosomes) (Huang and Reichardt, 2003; Reichardt, 2006; Marlin and Li, 2015), all processes requiring energetic input. Akt signaling has been reported to exhibit differential spatial and temporal profiles and subcellular domains of activation in response to epidermal growth factor in non-neuronal cells (Miura et al., 2014). The subcellular localization of PI3K-Akt signaling and bioenergetic sources utilized during TrkA signaling are not known. In this manuscript we present evidence of different spatio-temporal contributions of mitochondrial oxidative phosphorylation and glycolysis in the initiation and maintenance of NGF-TrkA signaling along embryonic sensory axons focusing on the phosphorylation of the PI3K-Akt signaling axis.

## Materials and Methods

### Neuron Culturing

Fertilized chicken eggs were obtained from Charles River Laboratories (#10100326). Dissociated primary embryonic day 7 chicken embryo neurons were prepared and cultured as previously detailed (Lelkes et al., 2005). Briefly, culturing was performed on coverslips coated with laminin (25 μg/mL: Life Technologies, #23017-015) in F12H medium. Unless otherwise noted, F12H medium (Thermo Fisher Scientific, # 11765047) was supplemented with 20 ng/mL NGF (R&D Systems, #256-GF-100; see Lelkes et al., 2005 for complete supplement formulation). Experiments involving acute NGF treatment (20 ng/mL) were performed using cultures raised in the absence of NGF for 24 h, see the Results for a rationale and description of the experimental approach. For experiment involving inhibition of glycolysis, at the time of experimental treatment the medium was switched to either control DMEM medium lacking glucose (Gibco, #11966025) and supplemented with 10 mM D-glucose (Sigma, #G7021), 10 mM Hepes (Fisher BioReagents, #BP299-100), 1% PSF (penicillin-streptomycin mixture; Thermo Fisher Scientific, #BW17745E) and 1 mM NaPyruvate (Gibco, #11360-070) or GIM (Glycolysis Inhibition Medium) prepared using the same formulation but replacing D-glucose with 10 mM 2-deoxyglucose (Sigma, #D8375). Antimycin-A was Sigma (#A8674). Neurons were cultured for 24 h prior to use in experiments.

### Immunocytochemistry

**FIGURE 1:**
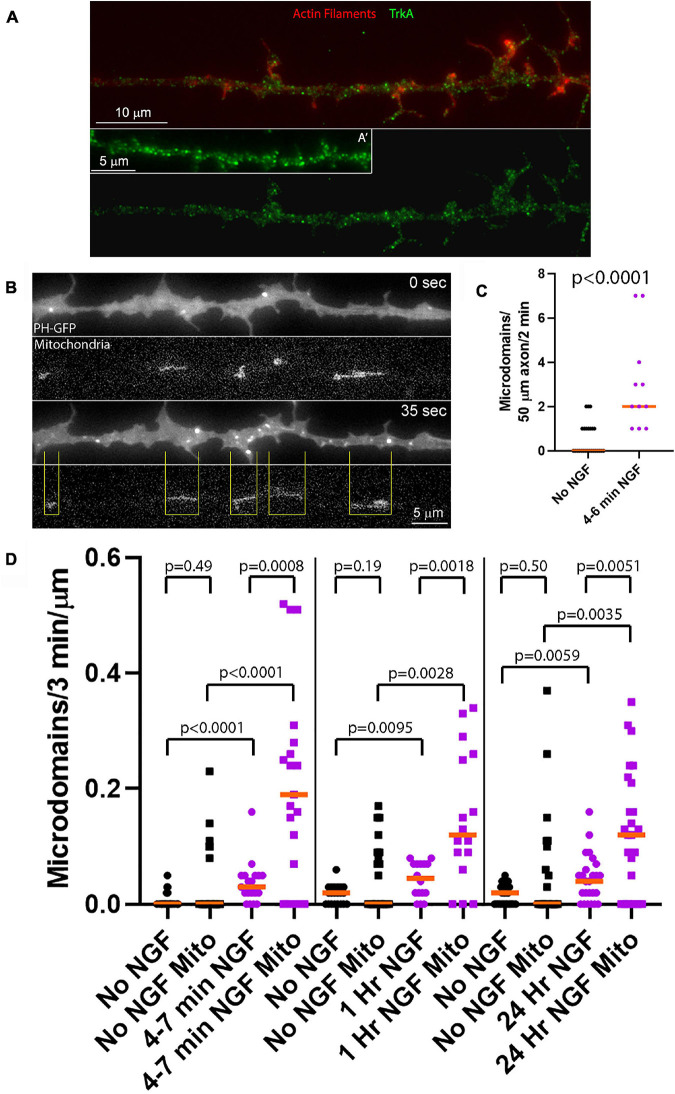
NGF preferentially increases the rate of formation of PIP3 microdomains in axon segments populated by mitochondria. **(A)** Examples of the distribution of the TrkA receptor in the membranes of sensory axons as determined by immunostaining with an antibody to the extracellular domain of TrkA under non-permeabilized conditions as in Gallo et al. (1997) and Ketschek and Gallo (2010). **(A′)** shows an example of an additional axon. Actin filaments were visualized using phalloidin. **(B)** Example of an NGF treated axon (4–7 min) expressing PH-GFP and with mitochondria labeled using mitochondrially targeted DsRed. At 0 s, representative of 4 min after NGF treatment, a few microdomains are visible as reflected by localized accumulation of PH-GFP. At 35 s a burst of microdomain formation has occurred. The yellow lines denote the positioning of mitochondria relative to the microdomains and define axon segments populated by mitochondria. Note that the majority of microdomains are formed in axon segments populated by mitochondria. **(C)** Quantification of the rate of microdomain formation per unit length/unit time of axon at 4–6 min after NGF treatment. Mann-Whitney test. **(D)** Analysis of the rate of microdomain formation in axon segments populated by mitochondria (Mito) and those lacking mitochondria as a function of time after NGF treatment. For each time point after NGF treatment (4–7 min, 1 and 24 h) time matched no NGF treatment control groups are shown. The rate of microdomain formation is normalized to the unit length of micron as mitochondria differ in length and the segments not populated by mitochondria similarly vary in length. The top row of *p*-values (shorter brackets) report on within NGF treatment group comparing axon segments populated by mitochondria to those that did not. The rest of the *p*-values report on comparisons within axon segments populated by mitochondria or not and between NGF treatment conditions. Mann-Whitney tests throughout.

Dissociated neurons were fixed with 4% paraformaldehyde (Electron Microscopy Sciences Cat# 15710) and 5% sucrose (Thermo Fisher Scientific Cat# S5-500) in PBS for 15 min at room temperature and blocked for 30 min with 10% Goat Serum (Sigma Cat# G9023) in PBS with 0.1% of Triton X-100 (Sigma Cat# 9002-93-1) (GST). Samples were incubated for 1 h with primary antibody against Phospho-Akt (Ser473) (Cell Signaling Technology Cat# 4060, RRID:AB_2315049), Phospho-Akt (Thr308) (Cell Signaling Technology Cat# 9275, RRID:AB_329828) and pan-AKT antibody (Abcam Cat# ab8805, RRID:AB_306791) diluted in GST (1:200) at room temperature and washed with PBS. Next, fluorescently labeled secondary antibody Anti-Rabbit IgG (whole molecule)-TRITC (Sigma-Aldrich Cat# T6778, RRID:AB_261740) and phalloidin Alexa 488 (1:20, Invitrogen Cat# A12379), were applied for an additional hour, at room temperature. To reveal axon morphology, in some experiments, axons were labeled with anti-α-tubulin conjugated to FITC (DM1A; 1:100, Sigma #F2168, RRID:AB_476967). After secondary antibody incubation, samples were washed with PBS and deionized water and mounted with Vectashield mounting media (Vector Cat# H-1000). Examples of TrkA staining shown in Figure 1A are from extant data sets initially published in Ketschek and Gallo (2010), and the relevant methods and antibody information are contained therein. The images shown here were not previously published.

### Plasmids and Transfection

The plasmids expressing PH-GFP and Mitochondrially targeted DsRed were prepared and used as described in Ketschek and Gallo (2010) and Spillane et al. (2013), respectively. Briefly, following dissociation the neuron suspension was transferred to a nucleofector cuvette containing 10 μg of Plasmid DNA, and electroporated using an Amaxa Nucleofector (program G-13; Lonza) and chicken transfection solution (Lonza). The electroporated neuronal suspension was then immediately transferred to a tube containing culturing media as described above prior to plating.

### Imaging

All imaging was performed using a Zeiss 200 M microscope equipped with an Orca-ER camera (Hamamatsu) in series with a PC workstation running Zeiss Axiovision software for image acquisition and analysis. Imaging of PH-GFP and mitochondrially targeted DsRed was performed as previously described in Ketschek and Gallo (2010) and Spillane et al. (2013), respectively. For live imaging, cultures were placed on a heated microscope stage (Zeiss temperable insert P with objective heater) for 10 min at a constant 39°C before and during imaging. Imaging was performed using a Zeiss Pan-Neofluar 100 X (1.3 numerical aperture). Time lapse imaging of PH-GFP and mitochondrially targeted DsRed were performed at 100x using 2 × 2 binning, 5 s inter-frame intervals, and minimal light (100 W mercury lamp). Still images of immunocytochemical staining were similarly obtained using the 100 x objective and phase contrast to visualize the axons. Due to the permeabilization required for immunocytochemical processing the phase contrast images are subpar but are only used to note the location of the axon shaft and not used to obtain data. In all cases, camera acquisition parameters were set so as not to obtain pixel saturation.

### Quantification of Akt Puncta and GFP-PH Microdomains

Puncta of phosphorylated Akt were manually counted along the distal 50 μm of axons excluding the growth cone. Supplementary Figure 1 shows an example and provides details of the line scan analysis used to determine puncta and their intensities. GFP-PH microdomains were analyzed as described in Ketschek and Gallo (2010) and follow the protocol for the analysis of axonal actin patches (Loudon et al., 2006) which show similar dynamics to PH-GFP microdomains (Ketschek and Gallo, 2010). The formation of a PIP3 microdomain was scored when the PH-GFP signal locally increased above a threshold of greater than 20% of the average cytoplasmic baseline signal wherein no local peaks were detected by line scan analysis, in the same way as shown for pAkt puncta in Supplementary Figure 1. The formation of a new microdomain in the same location as a prior one was only scored when the local intensity of the initial rise in the PH-GFP signal fell back to below 20% of the average cytoplasmic baseline signal for at least one frame.

### Statistical Analysis and Study Design

All data was analyzed using Instat software (GraphPad Software Inc.). Determination of the normalcy of data sets was performed using the Kolmogorov and Smirnov test. Normal data sets were analyzed using the Welch *t*-test for independent groups or the paired *t*-test for before-after treatment experimental designs. If non-normal data sets were present then non-parametric analysis was used (Mann-Whitney test). For multiple comparison tests within experimental designs parametric Bonferroni or non-parametric Dunn’s *post hoc* tests were used according to the normalcy of the data sets. One or two tailed *p*-values are reported based on whether the hypothesis predicted the directionality of the expected difference in mean or median, respectively. Graphs were generated using Prism (GraphPad Software Inc.). For presentation purposes, normally distributed data sets are shown using the mean and SEM and non-normally distributed data sets are shown as individual data points with the median noted.

All immunocytochemical data sets were acquired from at least triplicate cultures generated from ganglia harvested from multiple chicken embryos. All immunocytochemical samples within an experiment (control and experimental groups) were processed in parallel and imaged on the same day using the exact same acquisition parameters. For live imaging experiments using a before-after NGF treatment paradigm, each data point was obtained from a single neuron in a single culture. The full data set is thus representative of multiple cultures from multiple days.

## Results

### NGF-Induced PI3K Signaling Is Most Pronounced in Axon Segments Populated by Mitochondria

We have previously detailed that NGF induces the formation of localized actin filament patches that serve as precursors to the emergence of filopodia, and that actin patch formation is dependent on PI3K-Akt signaling and the sites of formation of actin patches colocalize with microdomains of phosphatidylinositol (3,4,5)-trisphosphate (PIP3) formation, the product of PI3K activity (Ketschek and Gallo, 2010). Sites of NGF-induced axonal actin patch and filopodia formation strongly correlate with the positioning of mitochondria along axons (Ketschek and Gallo, 2010; Spillane et al., 2013; Armijo-Weingart et al., 2019) and mitochondria respiration is required for actin patch formation and dynamics (Ketschek and Gallo, 2010; Sainath et al., 2017a). Therefore, we sought to determine if NGF-induced PIP3 microdomains along axons similarly correlate with sites populated by axonal mitochondria. To track PIP3 microdomain formation along axons we expressed the PIP3 binding pleckstrin homology (PH) domain of Akt tagged with GFP (Kontos et al., 1998) as in our prior work (PH-GFP; Ketschek and Gallo, 2010). We acknowledge that PI3K activity results in the generation of PIP3 that following dephosphorylation by polyphosphate 5-phosphatases results in the formation of PtdIns (3,4)P_2_ (Eramo and Mitchell, 2016) and both lipids are bound by the PH-domain of Akt (Lemmon et al., 2002). In the following text, we, however, use the terminology of PIP3 microdomains as in our prior work (Ketschek and Gallo, 2010) to refer to initial step in PI3K lipid phosphorylation based signaling. We have previously shown that pharmacological inhibition of PI3K suppresses detection of PIP3 microdomains reported by PH-GFP (Ketschek and Gallo, 2010). The term PIP3 microdomains thus refers to sites of PI3K activity.

In order to study the effects of acute treatment with NGF on a population of neurons responsive to NGF but naïve to NGF treatment, we cultured embryonic day 7 chicken embryo sensory neurons in the absence of NGF for 24 h on laminin coated substrata. Acute treatment with NGF means that cultures were raised for 24 h in no NGF conditions prior to treatment with NGF for the specified time period. This is a culturing model system wherein only the NGF responsive TrkA expressing population of neurons in sensory ganglia is able to survive and extend axons (Guan et al., 2003). We have used this experimental model system throughout our prior work addressing the effects of acute NGF treatment (Ketschek and Gallo, 2010; Spillane et al., 2012, 2013; Silver et al., 2014) and previously shown that NGF induces Akt phosphorylation and downstream phosphorylation of S6 in axons (Silver et al., 2014). Neurotrophins induce strong activation of Trk receptors and PI3K activity by 2–5 min of treatment (Kaplan et al., 1991; Soltoff et al., 1992). As previously described, TrkA receptors in the plasma membrane, detected with an antibody to the extracellular domain of TrKA in non-permeabilized samples, are widely distributed along the axonal population used in this study appearing as puncta all along the length of the axon (Figure 1A; Gallo et al., 1997; Ketschek and Gallo, 2010). At 4–6 min of NGF treatment increased the rate of PIP3 microdomain formation per unit length of distal axon (Figures 1B,C). Analysis of PIP3 microdomains along the axons of neurons with fluorescently labeled mitochondria showed that at 4–7 min after NGF treatment there was an increase in the rate of formation of PIP3 microdomains within axon segments populated by mitochondria, and also those not containing mitochondria, relative to axons not treated with NGF (Figures 1B,D). Axon segments populated by mitochondria were defined as the stretch of the axon encompassed between the distal and proximal ends of mitochondria (Figure 1B). However, after NGF treatment axon segments populated by mitochondria generated microdomains at median rates approximately 530% greater than those lacking mitochondria. Thus, these observations indicate that axon segments populated by mitochondria exhibit higher levels of PI3K signaling during the initial response to NGF treatment than those that do not contain mitochondria.

We next addressed PIP3 microdomain formation at 1 and 24 h after treatment with NGF, reflective of the later stages of NGF signaling when the amplitude of signaling has decreased to a lower level than during the initial phase following acute treatment with NGF but remaining above baseline no NGF treatment levels (Kaplan et al., 1991; Soltoff et al., 1992). As observed during the initial phase of signaling, in NGF treatment conditions segments of axons populated by mitochondria exhibited higher rates of PIP3 microdomain formation than those lacking mitochondria at both of these delayed time points (Figure 1D). Segments of axons not populated by mitochondria also exhibited increased rates of PIP3 microdomain formation (Figure 1D). At 1 and 24 h of NGF treatment, starting at 24 h of culturing in no NGF, the median rates of microdomain formation in axon segments populated by mitochondria relative to those not populated by mitochondria were elevated by 167 and 200%, respectively (Figure 1D), in contrast to the initial 530% difference at 4–7 min of treatment and consistent with decreased NGF signaling at these later stages. The data indicate that NGF signaling at later stages continues to elicit higher net levels of PI3K activity resulting in increased rates of PIP3 microdomain formation and that the effect of NGF is most pronounced at sites populated by mitochondria. In the absence of NGF signaling there was no difference in the median rate of microdomain formation as a function of mitochondria positioning (Figure 1D), although at all-time points axonal segments populated by mitochondria tended to exhibit maximal values in a range greater than those not containing mitochondria.

### Phosphorylation of Akt at T308 but Not S473 Localizes With Axon Segments Populated by Mitochondria

**FIGURE 2:**
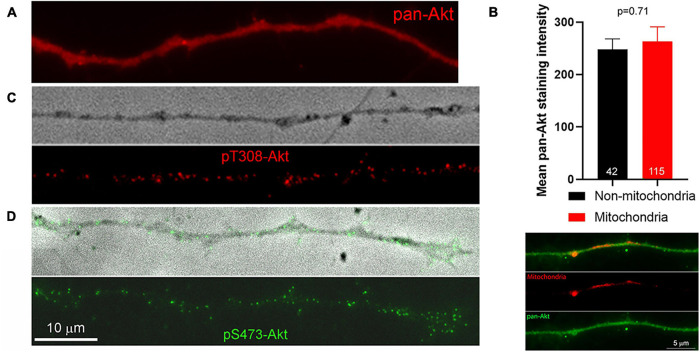
Distribution of pan-Akt, pT308-Akt and pS473-Akt. **(A)** Top panel shows an example of an axon stained with a pan-Akt antibody. The distribution is uniform. **(B)** Graph showing the mean staining intensity of pan-Akt staining in axons (*n* = 42) in segments devoid of mitochondria and those populated by mitochondria. An example of an axon stained with anti-pan-Akt and containing labeled mitochondria is shown below the graph. **(C)** Example of pT308-Akt staining along an axon treated with NGF for 10 min. The top panel shows a phase contrast image of the axon. **(D)** Example of pS473-Akt staining along an axon treated with NGF for 10 min. The top panel shows a phase contrast image of the axon with overlaid pS473-Akt. The scale bar in **(D)** also applies to **(A,C)**.

PIP3 generated through the activity of PI3K results in the targeting of Akt to the membrane through the binding of the PH domain of Akt to PIP3, and subsequent phosphorylation of Akt at T308 and S473 by PDK1 and mTORC2, respectively (Fayard et al., 2010). Full activation of Akt kinase activity is accomplished by di-phosphorylation at both residues, although Akt phosphorylated only at T308 exhibits some enhancement of kinase activity. As there are no biosensors for tracking the phosphorylation state of Akt, we used immunocytochemistry to gain insights into the spatio-temporal profile of Akt activation along axons in response to NGF (as previously used to measure increases and decreases in net levels of phosphorylated Akt along axons, Silver et al., 2014). We first considered the distribution of Akt, independent of its phosphorylation, in axons. Akt was uniformly distributed along the axons of neurons cultured in NGF and we did not observe any preferential association of Akt in axon segments populated by mitochondria (Figures 2A,B). The staining pattern for both pT308-Akt and pS473-Akt along the axons of neurons cultured in NGF was punctate, indicative of localized sites of phosphorylation (Figures 2C,D).

**FIGURE 3:**
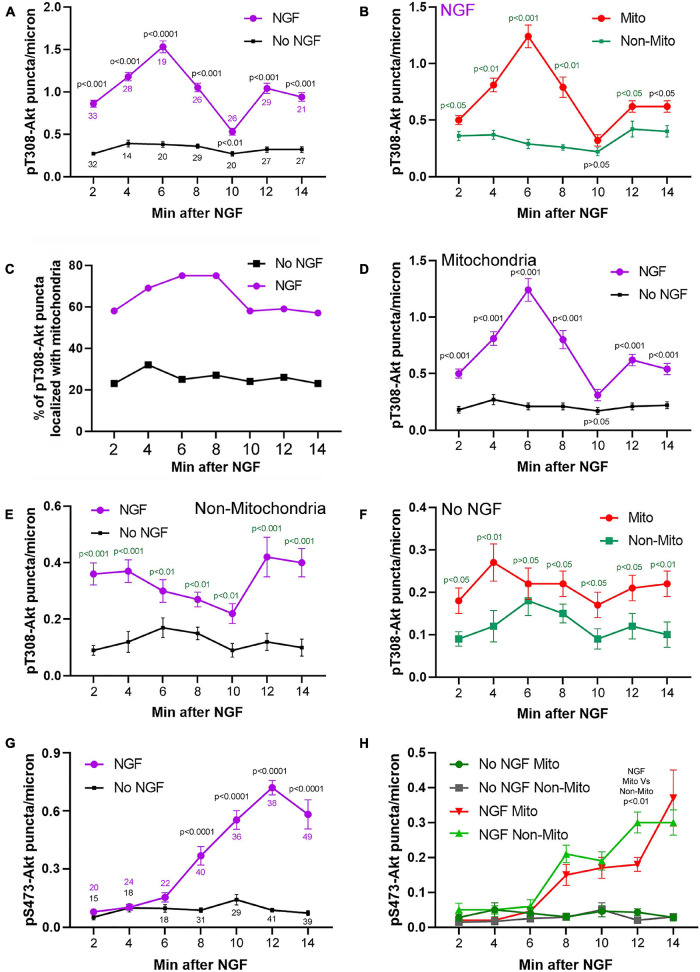
Time course and relationship of pT308-Akt and pS473-Akt to mitochondria positioning during the early phase (2–14 min) of NGF treatment. **(A)** Levels of pT308-Akt along the distal 50 mm of axon as a function of time after treatment with NGF or vehicle (no NGF). Throughout the figure data are expressed as the number of puncta/micron. Sample sizes (*n* = number of axons) at each time point are shown color coded for the respective group. **(B–F)** Provide further analysis of this data set. **(B)** Density of pT308-Akt puncta after treatment with NGF in axon segments populated by mitochondria (Mito) and those not containing mitochondria (Non-Mito). These data are extracted from the NGF treatment group in **(A)**. **(C)** Percent of pT308-Akt puncta that localized with mitochondria as a function of time after NGF treatment and no NGF conditions. These data are extracted from the NGF treatment group in **(A)**. **(D)** Comparison of the density of puncta of pT308-Akt in axon segments populated by mitochondria between NGF and no NGF treatment conditions. These data are extracted from the groups in **(A)**. **(E)** Comparison of the density puncta of pT308-Akt in axons segments not containing mitochondria as a function of time after NGF and no NGF treatment conditions. These data are extracted from the groups in **(A)**. **(F)** Comparison of the density of pT308-Akt puncta in the no NGF treatment condition in axon segments populated by mitochondria and those not containing mitochondria. These data are extracted from the no NGF treatment group in **(A)**. **(G)** Analysis of the levels of pS473-Akt in axons, as in **(A)**, as a function of time after treatment with NGF or no NGF. Sample sizes (*n* = number of axons) at each time point are shown color coded for the respective group. **(H)** Analysis of the density of puncta of pS473-Akt in axon segments populated by mitochondria and those not containing mitochondria in both the NGF and no NGF treatment conditions. These data are extracted from the NGF treatment group in **(G)**. Throughout the figure, time matched *post hoc* comparisons are shown.

The localization and degree of pT308-Akt and pS473-Akt labeling in the axons of neurons expressing mitochondrially targeted DsRed (as in Spillane et al., 2013) was then determined during the first 14 min of NGF-induced signaling. The relative levels of pT308-Akt signaling, reflected in the density of puncta per unit length of axon, increased by 2 min of NGF treatment and reached a peak within 6 min of treatment (Figure 3A). Analysis of the staining intensity of puncta of pT308-Akt did not reveal any difference after NGF treatment (Supplementary Figure 1). Hereafter, the density of puncta per micron of axon of phosphorylated Akt is thus used as the main metric for tracking the levels and position of phosphorylated pT308-Akt.

Axon segments populated by mitochondria accounted for the majority of the increase in density of pT308-Akt puncta during the initial 14 min of signaling (Figures 3B,C and Supplementary Figure 2A) and the density of pT308-Akt puncta in axon segments populated by mitochondria was highest at 6 min after NGF (Figure 3B and Supplementary Figure 2A). Considering that in these sensory axons mitochondria occupy approximately 15% of the area (Ketschek and Gallo, 2010), the localization of 60–80% of pT308-Akt puncta in axon segments populated by mitochondria represents a strong localization to these sub-axonal domains (Figure 3C). Both axon segments populated by mitochondria or devoid of mitochondria mitochondria exhibited an increase in the density of pT308-Akt puncta as soon as 2 min after NGF treatment (Figures 3D,E), but the peak in signaling observed in axon segments populated by mitochondria was not evident in segments not containing mitochondria (Figure 3E). The density of pT308-Akt puncta was also elevated in axon segments populated by mitochondria in the absence of NGF treatment (Figure 3F), indicating that even in the absence of NGF sites populated by mitochondria exhibit elevated levels of pT308-Akt. In no NGF conditions approximately 23–35% of pT308-Akt puncta colocalized with mitochondria at all-time points (Figure 3C), closer to but greater than the percentage of area covered by mitochondria (15%).

NGF treatment increased the density of pS473-Akt puncta within axons (Figures 2D, 3G show an example of axonal pS473-Akt staining). The increase in the density of pS473-Akt puncta became detectable at 8 min after NGF treatment, following the peak in pT308-Akt levels (Figure 3A). Analysis of the staining intensity of puncta of pS473-Akt did not reveal any difference after NGF treatment (Supplementary Figure 1), and thus as with pT308-Akt the density of pS473-Akt puncta was used as the main metric throughout. The density of pS473-Akt puncta following NGF treatment did not exhibit differences between axon segments populated by mitochondria and those not containing mitochondria (Figure 3H). Similarly, in the absence of NGF treatment there was no difference in the density of pS473-Akt puncta in axon segments populated by mitochondria and those not containing mitochondria (Figure 3H). The data thus indicate that phosphorylation of Akt at T308 in axons commences as soon as 2 min after NGF treatment and that axon segments populated by mitochondria experience higher levels of T308 phosphorylation. In contrast, the subsequent phosphorylation at S473, following the peak in T308 phosphorylation, does not correlate with mitochondria positioning along axons.

### Oxidative Phosphorylation (OxP) but Not Glycolysis Is Required for the Initial Phosphorylation of Akt at T308

In embryonic neurons both OxP and glycolysis contribute to ATP levels with relative contributions varying between neurons (reviewed in Gallo, 2020). In the population of neurons used in the current study, measurement of the ATP/ADP ratio showed that OxP [inhibited using antimycin-A (AA)] and glycolysis (inhibited by exchanging to medium containing no glucose and supplemented with 2-deoxyglucose to inhibit glycolysis and with pyruvate to maintain mitochondrial respiratory function, Glycolysis Inhibition Medium; GIM) both contribute approximately equally to the ATP/ADP ratio and inhibition of both has additive effects (Ketschek et al., 2021). While mitochondria occupy specific sites along the axon glycolytic enzymes show relatively even distributions within axons (Supplementary Figure 3).

**FIGURE 4:**
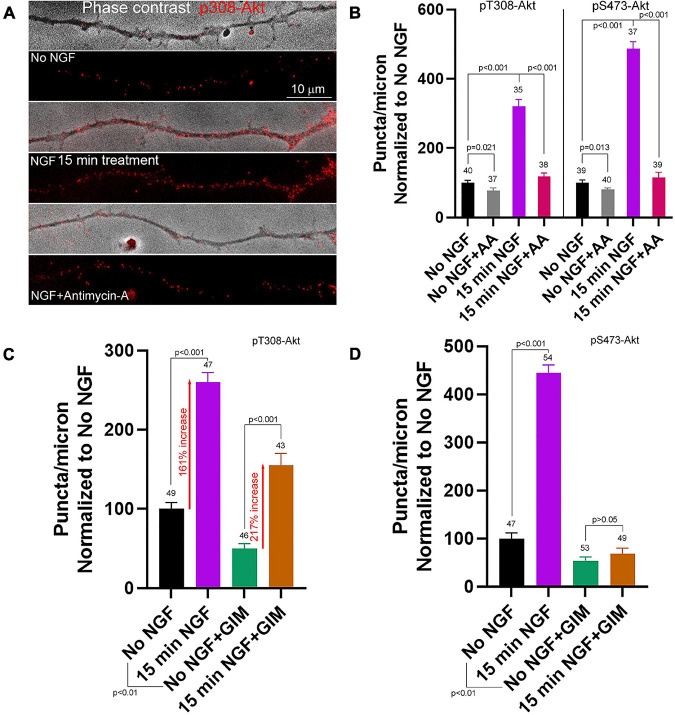
Contributions of oxidative phosphorylation and glycolysis to the early phase of NGF driven pT308-Akt and pS473-Akt phosphorylation. **(A)** Examples of pT308-Akt staining in axons (shown in overlay with phase contrast images) with 15 min treatments of no NGF, NGF or NGF with antimycin-A (AA). **(B)** Quantification of the density of pT308-Akt and pS473-Akt puncta following treatment with AA under no NGF and NGF 15 min treatment conditions. **(C)** Effects of inhibition of glycolysis using glycolysis inhibition medium (GIM) or control medium exchange on the density pT308-Akt puncta. GIM decreased the baseline density of pT308-Akt puncta in the absence of NGF, but NGF treatment elevated the density to a similar fold increase with both GIM and control medium (percent increase relative to the corresponding baseline is denoted). **(D)** GIM treatment alone decreased the baseline density of pS473-Akt puncta in no NGF treatment conditions and blocked the NGF induced increase in the density of pS473-Akt puncta.

The higher density of T308-Akt puncta associated with mitochondria led us to test the hypothesis that mitochondrial OxP might be required for this initial step of Akt signaling. Inhibition of OxP using AA (10 min pretreatment prior to NGF) blocked the NGF induced increase in the density of pT308-Akt puncta within distal axons (Figures 4A,B). Similarly, AA also blocked the NGF-induced increase in the density of pS473-Akt puncta (Figure 4B and Supplementary Figure 4A). Analysis of the spatial relationship of pT308-Akt puncta in relation to mitochondria showed that at 15 min of NGF treatment axon segments populated by mitochondria exhibited approximately 60% greater density of pT308-Akt puncta than those not containing mitochondria (Supplementary Figures 5A,C), consistent with the differences noted at 10–12 min of NGF treatment (Figure 3B). Consideration of the effects of AA treatment on the density of pT308-Akt puncta as a function of mitochondria positioning along axons showed that AA treatment suppressed the levels of pT308-Akt similarly in axon segments populated by or devoid of mitochondria (Supplementary Figures 5B,C).

In contrast, inhibition of glycolysis, using GIM exchange 15 min prior to NGF treatment did not block the NGF-induced increase in the density of pT308-Akt puncta along axons (Figure 4C and Supplementary Figure 4B), although GIM suppresses the ATP/ADP ratio by 15 min after treatment (Ketschek et al., 2021). GIM treatment alone decreased the baseline density of pT308-Akt puncta in the absence of NGF treatment. However, the magnitude of the increase in the density of pT308-Akt puncta after NGF treatment in GIM relative to the GIM treatment alone baseline was comparable to that in control medium (Figure 4C), indicating the magnitude of the response to NGF treatment in GIM does not differ from that in control medium, in sharp contrast to treatment with AA (Figure 4A). Similar to the treatment of GIM on the density of pT308-Akt puncta in the absence of NGF, GIM treatment decreased the density of pS473-Akt puncta along axons (Figure 4D). In contrast to pT308-Akt, GIM prevented the NGF-induced increase in the density of pS473-Akt puncta after 15 min of treatment (Figure 4D), a time point when the density of pS473-Akt puncta in response to NGF treatment have risen considerably (Figure 3G). The data thus indicate that the increases in phosphorylation of Akt the T308 and S473 induced by acute treatment with NGF require OxP, but S473 and not T308 phosphorylation also requires glycolysis.

### Bioenergetic Requirements of Steady State NGF Phosphorylation of Akt

**FIGURE 5:**
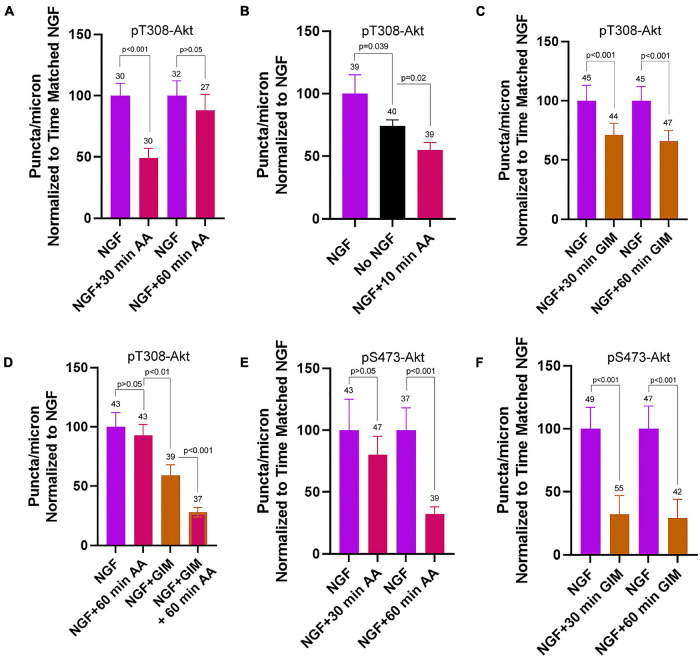
Contributions of oxidative phosphorylation and glycolysis to steady state NGF (24 h treatment) driven pT308-Akt and pS473-Akt phosphorylation. **(A)** Effects of a 30 and 60 min treatment with antimycin-A on the density of pT308-Akt puncta in axons. A 30 min treatment decreased the density that, however, restored to the time matched control levels by 60 min. **(B)** A 10 min treatment with AA decreased the density of pT308-Akt punctato a similar extent as a 30 min treatment **(A)**, and below those present in no NGF conditions. **(C)** GIM treatment for 30 and 60 min similarly decreased the density of pT308-Aktpuncta. **(D)** The restoration of the density of pT308-Akt punctato baseline after a 60 min treatment with AA is prevented by cotreatment with GIM. **(E)** Treatment with AA decreases the density of pS473-Akt puncta after a 60 min treatment but not a 30 min treatment. **(F)** Both 30 and 60 min treatments with GIM similarly decrease the density of pS473-Akt puncta.

After an initial high amplitude signaling period the levels of PI3K and Akt activity decline to a maintained steady state while in the continued presence of NGF (Kaplan et al., 1991; Soltoff et al., 1992). Here the term steady state refers to neurons cultured in the continuous presence of NGF from the time of culturing and used in experiments at 24 h of culturing. To assess the contribution of OxP to the maintenance of steady state levels of T308-Akt signaling we determined the effects of a 30 and 60 min treatment with AA on neurons cultured for 24 h in NGF. A 30 min treatment with AA decreased the density of pT308-Akt puncta along axons by approximately 50% (Figure 5A and Supplementary Figure 6A). The effect of AA treatment on the density of pT308-Akt puncta along axons was evident by 10 min of AA treatment and AA resulted in a greater suppression of the density of pT308-Akt puncta than the absence of NGF treatment (Figure 5B). Unexpectedly, by 60 min the density of pT308-Akt puncta had returned to baseline levels (Figure 5A and Supplementary Figure 6A). Inhibition of glycolysis by exchange of the medium with GIM resulted in a decrease in the density of pT308-Akt puncta along axons at both 30 and 60 min of treatment (Figure 5C and Supplementary Figure 6B). To determine if the return to baseline at 60 min of treatment with AA might be due to compensation through glycolysis we treated with GIM in the context of the same experimental design as in Figure 5A. Co-treatment with AA and GIM blocked the return of the density of pT308-Akt puncta to baseline levels following a 60 min AA treatment (Figure 5D and Supplementary Figure 6B). In GIM and AA treated samples measurements of the density of pT308-Akt puncta were lower than the GIM treatment condition alone (Figure 5D and Supplementary Figure 6B).

Inhibition of OxP using a 30 min treatment with AA did not alter the density of pS473-Akt puncta in axons (Figure 5E and Supplementary Figure 7A). However, a 60 min treatment resulted in an approximately 67% decrease (Figure 5E and Supplementary Figure 7A). Treatment with GIM for 30 or 60 min both decreased the density of pS473-Akt puncta along axons by approximately 75% (Figure 5F and Supplementary Figure 7B). These data indicate that the maintenance of pS473-Akt signaling in axons is more dependent on glycolytic function than OxP, but that both contribute to the maintenance of pS473-Akt signaling over a period of 1 h.

## Discussion

The relative contributions of mitochondrial OxP and glycolysis to the bioenergetics of axonal NGF signaling have not been previously addressed. In the experimental model system used in this study, mitochondria in distal sensory axons occupy specific domains and cover approximately 15% of the length of the axon (Ketschek and Gallo, 2010) while glycolytic enzymes are present in a mostly uniform distribution throughout axons (Supplementary Figure 3; Ketschek et al., 2021). We have previously reported evidence for local roles of mitochondrial OxP in regulating aspects axonal biology. Axonal segments populated by mitochondria are preferential sites for intra-axonal protein synthesis (Spillane et al., 2013; Sainath et al., 2017a) and also for the formation of axonal actin filament patches and the filopodia that emerge from these patches (Ketschek and Gallo, 2010; Sainath et al., 2017a), and mitochondrial OxP is required for mitochondria associated protein synthesis, actin patch/filopodia formation and the emergence of axon branches in response to NGF treatment (Ketschek and Gallo, 2010; Spillane et al., 2013; Sainath et al., 2017a). The data presented in this report add to these prior observations by showing that sites of NGF induced PI3K signaling (here termed PIP3 microdomains as in Ketschek and Gallo, 2010) and the subsequent downstream phosphorylation of Akt at T308, during the initial high amplitude signaling induced by NGF, are also preferentially associated with axon segments populated by mitochondria. Mitochondrial OxP, but not glycolysis, is required or the initiation of pT308-Akt signaling. In contrast to sites of PI3K activation and pT308-Akt, the activation of Akt through S473 phosphorylation was not found to be associated with mitochondria. The data also indicate that the net increase in Akt activity induced by NGF is due to an increase in localized sites of Akt phosphorylation, presenting as puncta in immunocytochemistry, without effects on the amount of phosphorylation at each site (as reflected by no changes in the intensity of the staining of individual sites/puncta of both pT308-Akt and pS473-Akt). The sites of PI3K activation and puncta of pT308-Akt likely correspond to clusters of TrkA receptors in the membrane, but this issue was not directly investigated. Thus, the data indicate that along sensory axons NGF increases the density of sites of initial Akt activation, reflected by pT308-Akt puncta, preferentially along axon segments populated by mitochondria. The suppression of pT308-Akt puncta by AA treatment at 15 min after NGF treatment along axon segments populated by mitochondria and those not containing mitochondria may reflect the redistribution of mitochondria that ensues during the first 10 min of NGF treatment (Armijo-Weingart et al., 2019) or the intracellular distribution of pT03-Akt puncta during this period. Consistent with the first suggestion, motile mitochondria can regulate aspects of synaptic function as they undergo transport (Sun et al., 2013), and thus may similarly regulate pT308-Akt. The relationship between motile mitochondria and sites of pT308-Akt will require further consideration.

Overall, the data suggest a hypothetical working model for the spatio-temporal initiation and spread of Akt signaling along axons, and the maintenance of the steady state during prolonged NGF signaling (summarized in visual form in Supplementary Figure 8). The data indicate that the initial phosphorylation of Akt at T308 occurs most prominently in axon segments populated by mitochondria which provide the required ATP through OxP. We suggest that his phosphorylation likely occurs at the plasma membrane or during early stages of endocytosis of TrkA-NGF couplets (Marlin and Li, 2015). Following phosphorylation at T308, Akt is considered to be associated with the ensuring NGF-TrKA signaling endosomes (Marlin and Li, 2015). We suggest that signaling endosomes recruit glycolytic enzymes that then serve to provide ATP for the phosphorylation of Akt at S473, as previously shown for the role of glycolysis in “on board” powering of the transport of axonal vesicles that also carry markers for endosomes (Zala et al., 2013; Hinckelmann et al., 2016). Thus, impairment of endocytosis would prevent the endosome associated phosphorylation of Akt at S473. Interestingly, the endocytosis of TrkA is dependent on PI3K signaling (York et al., 2000) as are other forms of receptor endocytosis (Bhattacharya et al., 2016). Some forms of endocytosis are dependent on actin filament dynamics (Chakrabarti et al., 2021) and PI3K-Akt signaling along axons in response to NGF promotes localized polymerization of actin patches preferentially in axonal segments populated by mitochondria (Ketschek and Gallo, 2010). Thus, the failure to phosphorylate Akt at T308 when OxP is suppressed is likely to also prevent TrkA endocytosis, and as shown by the data also the subsequent phosphorylation at S473. A prior example for internalization dependent activation of a signaling pathway by NGF-TrkA signaling is the activation of Erk signaling which is dependent on TrkA endocytosis (Zhang et al., 2000; Rakhit et al., 2001; Boutilier et al., 2008). Under steady state NGF conditions, due to the recycling of TrkA receptors to the plasma membrane (Chen et al., 2005; Ascaño et al., 2009, 2012; Yu et al., 2011; Diering et al., 2013), the process described above for the initial stage of NGF signaling would repeat. This could account for the delayed suppression of the levels of pS473-Akt in axons after inhibition of OxP but the faster suppression by inhibition of glycolysis. These aspects of the hypothetical mechanism are discussed further below. The issue of retrograde signaling is not addressed as here we focus on local actions of NGF on axons.

In the experimental model system used in this study, in the absence of NGF axons are growing on a laminin coated substratum and laminin signals through integrin receptors to promote the extension of NGF-responsive axons in the absence of NGF (Guan et al., 2003). The low levels of PI3K-Akt signaling present prior to NGF treatment are likely due to integrin signaling (Guidetti et al., 2015), although this was not specifically addressed. However, a 10 min inhibition of OxP in steady state NGF conditions suppressed pT308-Akt levels below those present in axons not treated with NGF, indicating that there are additional sources of OxP dependent pT308-Akt puncta than those activated by NGF. Upon treatment with NGF, which also hyperpolarizes the mitochondrial membrane potential along axons (Verburg and Hollenbeck, 2008), there is an increase in sites of PIP3 microdomain formation that is greatest in axon segments populated by mitochondria. The hyperpolarization of mitochondrial membrane potential by NGF requires PI3K signaling (Verburg and Hollenbeck, 2008). Thus, it is possible that a local feedforward loop is activated wherein NGF PI3K signaling promotes mitochondrial respiration that in turn promotes PI3K signaling locally within axon segments populated by mitochondria. PIP3 microdomains would then serve as scaffolds for the recruitment of Akt to the membrane and phosphorylation at T308 by PDK1. As the PIP3 microdomains are largely associated with mitochondria, the resulting phosphorylation of Akt at T308 is also similarly localized during the early phase of signaling.

In our investigation, the subsequent phosphorylation of Akt at S473 induced by NGF, presumably mediated by mTORC2, did not exhibit preferential localization with mitochondria and occurred with a similar temporal profile in axon segments populated by mitochondria and those not containing mitochondria. It is generally considered that mTORC2 is also localized to membranes through the binding of PI3K lipid products, but evidence indicates that the targeting may be at the plasma membrane or at endomembranes (Manning and Toker, 2017). The latter possibility is a better fit for the observation that S473 phosphorylation is not preferentially localized within axon segments populated by mitochondria and their associated PIP3 microdomains. NGF signaling induces the formation of signaling endosomes that then undergo intracellular redistribution (Marlin and Li, 2015). The data suggest the hypothesis that S473 phosphorylation may be occurring on signaling endosomes as they redistribute, but this possibility would need to be investigated empirically.

The imaging methods used in this study to address PIP3 localization have resolution limitations. The application of super resolution methods may reveal the presence of smaller PIP3 microdomains not visible using our approach. If the hypothesis that signaling endosomes are the sites of S473 Akt phosphorylation is valid, then super resolution imaging may be able to correlate endosomal markers with PI3K lipid products and phosphorylation of Akt at S473. However, inhibition of OxP also prevented S473 phosphorylation during the initial burst of NGF signaling. The requirement for OxP in S473 phosphorylation may be due to a requirement for OxP in the traffic mTORC2 to membranes, or inhibition of OxP may also be preventing the initial steps of TrkA activation upstream of PI3K signaling thereby shutting down subsequent signaling events, or in the endocytosis of TrkA receptors, issues not addressed in this study that will require future investigation.

Proteomic analysis of fast transport vesicles has found that glycolytic enzymes are associated with vesicles that also exhibit endosomal markers and that ATP production through glycolysis can occur on vesicles and powers their transport (Zala et al., 2013; Hinckelmann et al., 2016). Thus, glycolytic machinery is associated with endosomes. The observations that the initial NGF-induced S473 Akt phosphorylation is dependent on glycolysis and that suppression of glycolysis results in a faster decline in S473 levels than following suppression of OxP at steady state indicate a relationship between S473 phosphorylation and glycolysis. The data also suggest the hypothesis that S473 phosphorylation of Akt may occur on internalized Trk-NGF containing signalosomes.

Under conditions of steady state NGF signaling (treatment for 24 h) inhibition of OxP suppressed the levels of pT308-Akt by 30 min of treatment, but the levels recovered to baseline by 60 min, and the recovery required glycolysis. If the above consideration that OxP is required for the activation of NGF-TrkA signaling is valid, then under steady state conditions glycolysis may be able to compensate for the loss of OxP. This hypothetical compensatory mechanism has a precedence in the literature. Jang et al. (2016) report that under conditions of energy stress glycolytic enzymes are recruited to synaptic sites to compensate for decrease oxidative respiration or decreased mitochondrial numbers and function along neurites. Furthermore, Jang et al. (2016) also present evidence that glycolysis can power synaptic vesicle recycling when mitochondria function is impaired. The relevance of these observations to the current study is further emphasized by the prior demonstrations that sites of axonal filopodia and branch formation colocalize with mitochondria and accumulations of synaptic vesicles along axons (Courchet et al., 2013; Greif et al., 2013).

Inhibition of glycolysis also suppressed steady state pT308-Akt levels but by approximately 50% of the decrease observed with inhibition of OxP. These data indicate that although glycolysis is not required for the initial burst of pT308-Akt phosphorylation during the initial 15 min of NGF signaling, it does contribute to its maintenance during steady state signaling. The difference in the role of glycolysis will require further consideration but may reflect pools of internalized NGF-TrkA couplets in signalosomes, that may be more prevalent during steady state signaling than the initial phase of signaling, and these signalosomes may associate with the glycolytic apparatus as described for other fast transport vesicles (Zala et al., 2013; Hinckelmann et al., 2016). In contrast, the steady state levels of pS473-Akt dropped equally by approximately 75% with both a 30 and 60 min inhibition of glycolysis. The data thus indicate that the maintenance of pS473-Akt under steady state conditions of NGF signaling is more dependent on glycolysis, which we speculate may be occurring on internalized signalosomes.

In conclusion, this study indicates that OxP and glycolysis contribute differently to the activation of the PI3K-Akt signaling axis by NGF along sensory axons. The data suggest that the initial activation of PI3K and pT308-Akt signaling occurs preferentially in axon segments populated by mitochondria, and that OxP is required for the initial phosphorylation of Akt at T308 independent of glycolysis. These initial observations suggest that additional work will be required to dissect the relative contributions of OxP and glycolysis to signaling along axons, and that the two bioenergetic sources may contribute differentially depending on the time frame, and possibly intracellular sites, of the signaling. Whether similar bioenergetic requirements may apply to other ligand-receptor signaling systems remains an open question.

## Data Availability Statement

The raw data supporting the conclusions of this article will be made available by the authors, without undue reservation.

## Ethics Statement

The animal study was reviewed and approved by the Institutional Animal Care and Use Committee, Lewis Katz School of Medicine, Temple University.

## Author Contributions

GG designed the research, analyzed the data, and prepared the manuscript. RS designed, conducted the research, and analyzed the data. Both authors contributed to the article and approved the submitted version.

## Conflict of Interest

The authors declare that the research was conducted in the absence of any commercial or financial relationships that could be construed as a potential conflict of interest.

## Publisher’s Note

All claims expressed in this article are solely those of the authors and do not necessarily represent those of their affiliated organizations, or those of the publisher, the editors and the reviewers. Any product that may be evaluated in this article, or claim that may be made by its manufacturer, is not guaranteed or endorsed by the publisher.

## References

[B1] Armijo-Weingart L. Gallo G. (2017). It takes a village to raise a branch: cellular mechanisms of the initiation of axon collateral branches. *Mol. Cell.Neurosci.* 84 36–47. 10.1016/j.mcn.2017.03.007 28359843 PMC5617777

[B2] Armijo-Weingart L. Ketschek A. Sainath R. Pacheco A. Smith G. M. Gallo G. (2019). Neurotrophins induce fission of mitochondria along embryonic sensory axons. *Elife* 8:e49494. 10.7554/eLife.49494 31789589 PMC6887118

[B3] Ascaño M. Bodmer D. Kuruvilla R. (2012). Endocytic trafficking of neurotrophins in neural development. *Trends Cell Biol.* 22 266–273. 10.1016/j.tcb.2012.02.005 22444728 PMC3348440

[B4] Ascaño M. Richmond A. Borden P. Kuruvilla R. (2009). Axonal targeting of Trk receptors via transcytosis regulates sensitivity to neurotrophin responses. *J. Neurosci.* 29 11674–11685. 10.1523/JNEUROSCI.1542-09.2009 19759314 PMC2775807

[B5] Ashrafi G. Ryan T. A. (2017). Glucose metabolism in nerve terminals. *Curr. Opin. Neurobiol.* 45 156–161. 10.1016/j.conb.2017.03.007 28605677 PMC5675126

[B6] Bhattacharya S. McElhanon K. E. Gushchina L. V. Weisleder N. (2016). Role of phosphatidylinositol-4,5-bisphosphate 3-kinase signaling in vesicular trafficking. *Life Sci.* 167 39–45. 10.1016/j.lfs.2016.10.018 27760304 PMC5204450

[B7] Boutilier J. Ceni C. Pagdala P. C. Forgie A. Neet K. E. Barker P. A. (2008). Proneurotrophins require endocytosis and intracellular proteolysis to induce TrkA activation. *J. Biol. Chem.* 19 12709–12716. 10.1074/jbc.M710018200 18299325 PMC2442317

[B8] Chakrabarti R. Lee M. Higgs H. N. (2021). Multiple roles for actin in secretory and endocytic pathways. *Curr. Biol.* 31 R603–R618. 10.1016/j.cub.2021.03.038 34033793 PMC9759210

[B9] Chen Z. Y. Ieraci A. Tanowitz M. Lee F. S. (2005). A novel endocytic recycling signal distinguishes biological responses of Trk neurotrophin receptors. *Mol. Biol. Cell.* 16 5761–5772. 10.1091/mbc.e05-07-0651 16207814 PMC1289419

[B10] Courchet J. Lewis T. L. Jr. Lee S. Courchet V. Liou D. Y. Aizawa S. (2013). Terminal axon branching is regulated by the LKB1-NUAK1 kinase pathway via presynaptic mitochondrial capture. *Cell* 153 1510–1525. 10.1016/j.cell.2013.05.021 23791179 PMC3729210

[B11] Diering G. H. Numata Y. Fan S. Church J. Numata M. (2013). Endosomal acidification by Na^+^/H^+^ exchanger NHE5 regulates TrkA cell-surface targeting and NGF-induced PI3K signaling. *Mol. Biol. Cell.* 24 3435–3448. 10.1091/mbc.E12-06-0445 24006492 PMC3814139

[B12] Eramo M. J. Mitchell C. A. (2016). Regulation of PtdIns(3,4,5)P3/Akt signalling by inositol polyphosphate 5-phosphatases. *Biochem. Soc. Trans.* 44 240–252. 10.1042/BST20150214 26862211

[B13] Fayard E. Xue G. Parcellier A. Bozulic L. Hemmings B. A. (2010). “Protein kinase B (PKB/Akt), a key mediator of the PI3K signaling pathway,” in *Phosphoinositide 3-Kinase in Health and Disease. Current Topics in Microbiology and Immunology*, Vol. 346 eds Rommel C. Vanhaesebroeck B. Vogt P. (Berlin: Springer). 10.1007/82_2010_58 20517722

[B14] Gallo G. (2020). The bioenergetics of neuronal morphogenesis and regeneration: frontiers beyond the mitochondrion. *Dev. Neurobiol.* 80 263–276. 10.1002/dneu.22776 32750228 PMC7749811

[B15] Gallo G. Lefcort F. B. Letourneau P. C. (1997). The trkA receptor mediates growth cone turning toward a localized source of nerve growth factor. *J. Neurosci.* 17 5445–5454. 10.1523/JNEUROSCI.17-14-05445.1997 9204927 PMC6793814

[B16] Gallo G. Letourneau P. C. (2000). Neurotrophins and the dynamic regulation of the neuronal cytoskeleton. *J. Neurobiol.* 44 159–173. 10.1002/1097-4695(200008)44:2<159::aid-neu6<3.0.co;2-h 10934319

[B17] Greif K. F. Asabere N. Lutz G. J. Gallo G. (2013). Synaptotagmin-1 promotes the formation of axonal filopodia and branches along the developing axons of forebrain neurons. *Dev. Neurobiol.* 73 27–44. 10.1002/dneu.22033 22589224

[B18] Guan W. Puthenveedu M. A. Condic M. L. (2003). Sensory neuron subtypes have unique substratum preference and receptor expression before target innervation. *J. Neurosci.* 23 1781–1791. 10.1523/JNEUROSCI.23-05-01781.2003 12629182 PMC6741987

[B19] Guidetti G. F. Canobbio I. Torti M. (2015). PI3K/Akt in platelet integrin signaling and implications in thrombosis. *Adv. Biol. Regul.* 59 36–52. 10.1016/j.jbior.2015.06.001 26159296

[B20] Han S. M. Baig H. S. Hammarlund M. (2016). Mitochondria localize to injured axons to support regeneration. *Neuron* 92 1308–1323. 10.1016/j.neuron.2016.11.025 28009276 PMC5364819

[B21] Hinckelmann M. V. Virlogeux A. Niehage C. Poujol C. Choquet D. Hoflack B. (2016). Self-propelling vesicles define glycolysis as the minimal energy machinery for neuronal transport. *Nat. Commun.* 7:13233. 10.1038/ncomms13233 27775035 PMC5078996

[B22] Huang E. J. Reichardt L. F. (2003). Trk receptors: roles in neuronal signal transduction. *Annu. Rev. Biochem.* 72 609–642. 10.1146/annurev.biochem.72.121801.161629 12676795

[B23] Jang S. Nelson J. C. Bend E. G. Rodríguez-Laureano L. Tueros F. G. Cartagenova L. (2016). Glycolytic enzymes localize to synapses under energy stress to support synaptic function. *Neuron* 90 278–291. 10.1016/j.neuron.2016.03.011 27068791 PMC4840048

[B24] Kaplan D. R. Martin-Zanca D. Parada L. F. (1991). Tyrosine phosphorylation and tyrosine kinase activity of the trk proto-oncogene product induced by NGF. *Nature* 350 158–160. 1706478 10.1038/350158a0

[B25] Ketschek A. Gallo G. (2010). Nerve growth factor induces axonal filopodia through localized microdomains of phosphoinositide 3-kinase activity that drive the formation of cytoskeletal precursors to filopodia. *J. Neurosci.* 30 12185–12197. 10.1523/JNEUROSCI.1740-10.2010 20826681 PMC2944214

[B26] Ketschek A. Sainath R. Holland S. Gallo G. (2021). The axonal glycolytic pathway is required for sensory axon extension and growth cone dynamics. *J. Neurosci.* 41 6637–6651. 10.1523/JNEUROSCI.0321-21.2021 34252036 PMC8336710

[B27] Kontos C. D. Stauffer T. P. Yang W. P. York J. D. Huang L. Blanar M. A. (1998). Tyrosine 1101 of Tie2 is the major site of association of p85 and is required for activation of phosphatidylinositol 3-kinase and Akt. *Mol. Cell. Biol.* 18 4131–4140. 10.1128/MCB.18.7.4131 9632797 PMC108997

[B28] Lelkes P. I. Unsworth B. R. Saporta S. Cameron D. F. Gallo G. (2005). “Chapter 14 culture of neuroendocrine and neuronal cells for tissue engineering,” in *Culture of Cells of Tissue Engineering*, ed. Freshney R. I. (Hoboken, NJ: John Wiley & Sons). 10.1002/0471741817.ch14

[B29] Lemmon M. A. Ferguson K. M. Abrams C. S. (2002). Pleckstrin homology domains and the cytoskeleton. *FEBS Lett.* 513 71–76. 10.1016/s0014-5793(01)03243-4 11911883

[B30] Lewis T. L. Jr. Kwon S. K. Lee A. Shaw R. Polleux F. (2018). MFF-dependent mitochondrial fission regulates presynaptic release and axon branching by limiting axonal mitochondria size. *Nat. Commun.* 9:5008. 10.1038/s41467-018-07416-2 30479337 PMC6258764

[B31] Loudon R. P. Silver L. D. Yee H. F. Jr. Gallo G. (2006). RhoA-kinase and myosin II are required for the maintenance of growth cone polarity and guidance by nerve growth factor. *J. Neurobiol.* 66 847–867. 10.1002/neu.20258 16673385 PMC1525020

[B32] Manning B. D. Toker A. (2017). AKT/PKB signaling: navigating the network. *Cell* 169 381–405. 10.1016/j.cell.2017.04.001 28431241 PMC5546324

[B33] Marlin M. C. Li G. (2015). Biogenesis and function of the NGF/TrkA signaling endosome. *Int. Rev. Cell. Mol. Biol.* 314 239–257. 10.1016/bs.ircmb.2014.10.002 25619719 PMC4307610

[B34] Miura H. Matsuda M. Aoki K. (2014). Development of a FRET biosensor with high specificity for Akt. *Cell Struct. Funct.* 39 9–20. 10.1247/csf.13018 24212374

[B35] Morris R. L. Hollenbeck P. J. (1993). The regulation of bidirectional mitochondrial transport is coordinated with axonal outgrowth. *J. Cell Sci.* 104 917–927. 8314882 10.1242/jcs.104.3.917

[B36] Niewiadomska G. Mietelska-Porowska A. Mazurkiewicz M. (2011). The cholinergic system, nerve growth factor and the cytoskeleton. *Behav. Brain Res.* 221 515–526. 10.1016/j.bbr.2010.02.024 20170684

[B37] Pacelli C. Giguère N. Bourque M. J. Lévesque M. Slack R. S. Trudeau L. É (2015). Elevated mitochondrial bioenergetics and axonal arborization size are key contributors to the vulnerability of dopamine neurons. *Curr. Biol.* 25 2349–2360. 10.1016/j.cub.2015.07.050 26320949

[B38] Pacheco A. Gallo G. (2016). Actin filament-microtubule interactions in axon initiation and branching. *Brain Res. Bull.* 126 300–310. 10.1016/j.brainresbull.2016.07.013 27491623 PMC5518172

[B39] Patrón L. A. Zinsmaier K. E. (2016). Mitochondria on the road to power axonal regeneration. *Neuron* 92 1152–1154. 10.1016/j.neuron.2016.12.007 28009268

[B40] Rakhit S. Pyne S. Pyne N. J. (2001). Nerve growth factor stimulation of p42/p44 mitogen-activated protein kinase in PC12 cells: role of G(i/o), G protein-coupled receptor kinase 2, beta-arrestin I, and endocytic processing. *Mol. Pharmacol.* 60 63–70. 10.1124/mol.60.1.63 11408601

[B41] Rangaraju V. Lewis T. L. Jr. Hirabayashi Y. Bergami M. Motori E. Cartoni R. (2019). Pleiotropic mitochondria: the influence of mitochondria on neuronal development and disease. *J. Neurosci.* 39 8200–8208. 10.1523/JNEUROSCI.1157-19.2019 31619488 PMC6794931

[B42] Reichardt L. F. (2006). Neurotrophin-regulated signalling pathways. *Philos. Trans. R. Soc. Lond. B Biol. Sci.* 361 1545–1564. 10.1098/rstb.2006.1894 16939974 PMC1664664

[B43] Sainath R. Armijo-Weingart L. Ketscheck A. Xu Z. Li S. Gallo G. (2017b). Chondroitin sulfate proteoglycans negatively regulate the positioning of mitochondria and endoplasmic reticulum to distal axons. *Dev. Neurobiol.* 77 1351–1370. 10.1002/dneu.22535 28901718 PMC5693728

[B44] Sainath R. Ketschek A. Grandi L. Gallo G. (2017a). CSPGs inhibit axon branching by impairing mitochondria-dependent regulation of actin dynamics and axonal translation. *Dev. Neurobiol.* 77 454–473. 10.1002/dneu.22420 27429169 PMC5243930

[B45] Sheng Z. H. (2017). The interplay of axonal energy homeostasis and mitochondrial trafficking and anchoring. *Trends Cell Biol.* 27 403–416. 10.1016/j.tcb.2017.01.005 28228333 PMC5440189

[B46] Silver L. Michael J. V. Goldfinger L. E. Gallo G. (2014). Activation of PI3K and R-Ras signaling promotes the extension of sensory axons on inhibitory chondroitin sulfate proteoglycans. *Dev. Neurobiol.* 74 918–933. 10.1002/dneu.22174 24578264 PMC4235675

[B47] Smith G. M. Gallo G. (2018). The role of mitochondria in axon development and regeneration. *Dev. Neurobiol.* 78 221–237. 10.1002/dneu.22546 29030922 PMC5816701

[B48] Soltoff S. P. Rabin S. L. Cantley L. C. Kaplan D. R. (1992). Nerve growth factor promotes the activation of phosphatidylinositol 3-kinase and its association with the trk tyrosine kinase. *J. Biol. Chem.* 267 17472–17477. 1380963

[B49] Spillane M. Ketschek A. Donnelly C. J. Pacheco A. Twiss J. L. Gallo G. (2012). Nerve growth factor-induced formation of axonal filopodia and collateral branches involves the intra-axonal synthesis of regulators of the actin-nucleating Arp2/3 complex. *J. Neurosci.* 32 17671–17689. 10.1523/JNEUROSCI.1079-12.2012 23223289 PMC3596264

[B50] Spillane M. Ketschek A. Merianda T. T. Twiss J. L. Gallo G. (2013). Mitochondria coordinate sites of axon branching through localized intra-axonal protein synthesis. *Cell Rep.* 5 1564–1575. 10.1016/j.celrep.2013.11.022 24332852 PMC3947524

[B51] Sun T. Qiao H. Pan P. Y. Chen Y. Sheng Z. H. (2013). Motile axonal mitochondria contribute to the variability of presynaptic strength. *Cell Rep.* 4 413–419. 10.1016/j.celrep.2013.06.040 23891000 PMC3757511

[B52] Tao K. Matsuki N. Koyama R. (2014). AMP-activated protein kinase mediates activity-dependent axon branching by recruiting mitochondria to axon. *Dev. Neurobiol.* 74 557–573. 10.1002/dneu.22149 24218086

[B53] Verburg J. Hollenbeck P. J. (2008). Mitochondrial membrane potential in axons increases with local nerve growth factor or semaphorin signaling. *J. Neurosci.* 28 8306–8315. 10.1523/JNEUROSCI.2614-08.2008 18701693 PMC2597466

[B54] Wong H. H. Lin J. Q. Ströhl F. Roque C. G. Cioni J. M. Cagnetta R. (2017). RNA docking and local translation regulate site-specific axon remodeling in vivo. *Neuron* 95 852–868.e8. 10.1016/j.neuron.2017.07.016 28781168 PMC5563073

[B55] York R. D. Molliver D. C. Grewal S. S. Stenberg P. E. McCleskey E. W. Stork P. J. (2000). Role of phosphoinositide 3-kinase and endocytosis in nerve growth factor-induced extracellular signal-regulated kinase activation via Ras and Rap1. *Mol. Cell Biol.* 20 8069–8083. 10.1128/MCB.20.21.8069-8083.2000 11027277 PMC86417

[B56] Yu T. Calvo L. Anta B. López-Benito S. Southon E. Chao M. V. (2011). Regulation of trafficking of activated TrkA is critical for NGF-mediated functions. *Traffic* 12 521–534. 10.1111/j.1600-0854.2010.01156.x 21199218 PMC3547592

[B57] Zala D. Hinckelmann M. V. Yu H. Lyra da Cunha M. M. Liot G. Cordelières F. P. (2013). Vesicular glycolysis provides on-board energy for fast axonal transport. *Cell* 152 479–491. 10.1016/j.cell.2012.12.029 23374344

[B58] Zhang Y. Moheban D. B. Conway B. R. Bhattacharyya A. Segal R. A. (2000). Cell surface Trk receptors mediate NGF-induced survival while internalized receptors regulate NGF-induced differentiation. *J. Neurosci.* 20 5671–5678. 10.1523/JNEUROSCI.20-15-05671.2000 10908605 PMC6772538

[B59] Zhou B. Yu P. Lin M. Y. Sun T. Chen Y. Sheng Z. H. (2016). Facilitation of axon regeneration by enhancing mitochondrial transport and rescuing energy deficits. *J. Cell Biol.* 214 103–119. 10.1083/jcb.201605101 27268498 PMC4932375

[B60] Zhou F. Q. Zhou J. Dedhar S. Wu Y. H. Snider W. D. (2004). NGF-induced axon growth is mediated by localized inactivation of GSK-3beta and functions of the microtubule plus end binding protein APC. *Neuron* 42 897–912. 10.1016/j.neuron.2004.05.011 15207235

