# Supplementary material for: Bioenergetic Requirements and Spatiotemporal Profile of Nerve Growth Factor Induced PI3K-Akt Signaling Along Sensory Axons
Source: Front Mol Neurosci. 2021 Sep 24;14:726331. doi: 10.3389/fnmol.2021.726331 (PMC8497901; doi:10.3389/fnmol.2021.726331)
Supplement: Supplementary file 1 [file Image_1.pdf]

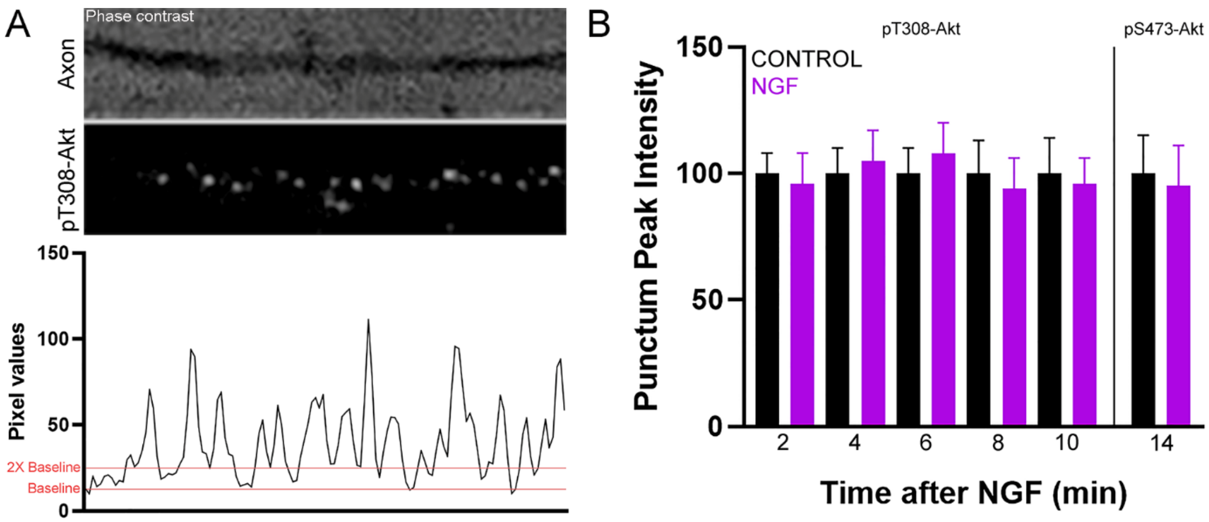

Supplemental Figure 1. Analysis of pAkt punctum density and intensity. **(A)** Example of line scan analysis of pAkt punctum intensity. The pictures show the phase contrast outline of the axon and the series of pT308-Akt puncta contained within the axon. The graph shows the pixel intensities derived from line scan analysis using Image J. Individual peak punctum intensity was determined by subtraction of the baseline intensity value from the maximal peak intensity value for each punctum in each axon (n=as in Figure 3A and G for pT308 and p473 respectively). Baseline was determined as the mean in regions of the axon not containing puncta. Peaks above twice the baseline intensity were considered to be reflective of pAkt puncta and included in the analysis. The slight visual off set between the line scan and the image is due to the necessity of running the line scan through the centers of the puncta which are not in a strictly linear relationship to one another when occurring in close vicinity. **(B)** No differences were found for punctum intensity within time point between time matched no NGF and NGF treatment for either pT308-Akt or pS473-Akt (Bonferroni multiple comparison tests within time point across no NGF and NGF treatment; n=number of axons as shown in Figure 3A and G for pT308 and pS473 respectively). The analysis for both phosphorylation sites was performed during times

after NGF treatment when the increase in puncta density was high during the initial 14 min treatment window. For each time point the intensities are normalized to the time matched control.

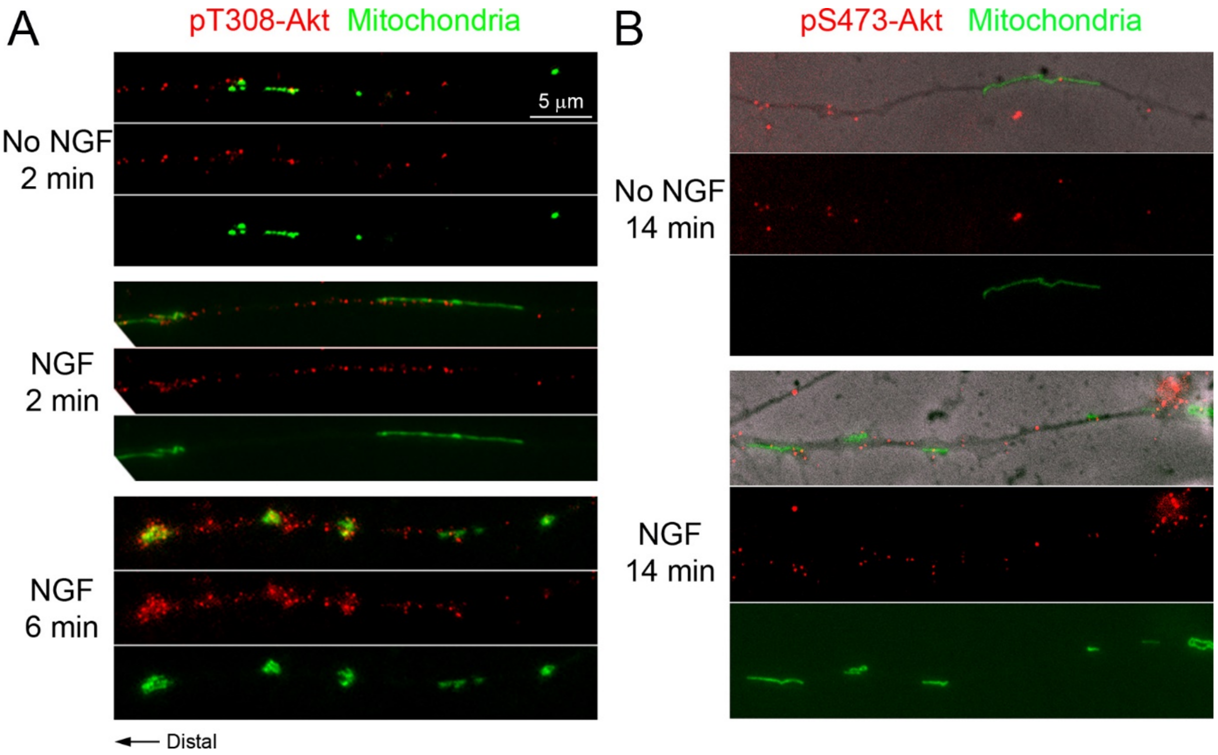

Supplemental Figure 2. Examples of the localization of pAkt along axons with labeled mitochondria as a function of treatment with NGF. **(A)** Examples of the distribution and levels of pT308-Akt puncta along axons. As indicated by the quantification of puncta density in Figure 3A by 2 min of NGF treatment there is an increase in puncta which only partially overlaps with mitochondria Figure 3B. By 6 min of NGF treatment the increase in puncta is more pronounced and the majority as associated with axon segments containing mitochondria. **(B)** Examples of the distribution and levels of pS473-Akt puncta along axons. Examples of time matched control and 14 min NGF treatment are shown. The phase contrast images are also presented as with pS473-Akt the puncta were often associated with the edges of the axon and protrusions. Although by 14 min of NGF treatment the density of puncta is increased (Figure 3G), the puncta do not show preferential localization within axon segments populated by mitochondria (Figure 3H). The scale bar in top leftmost panel applies to all panels. Distal is to the left in all panels.

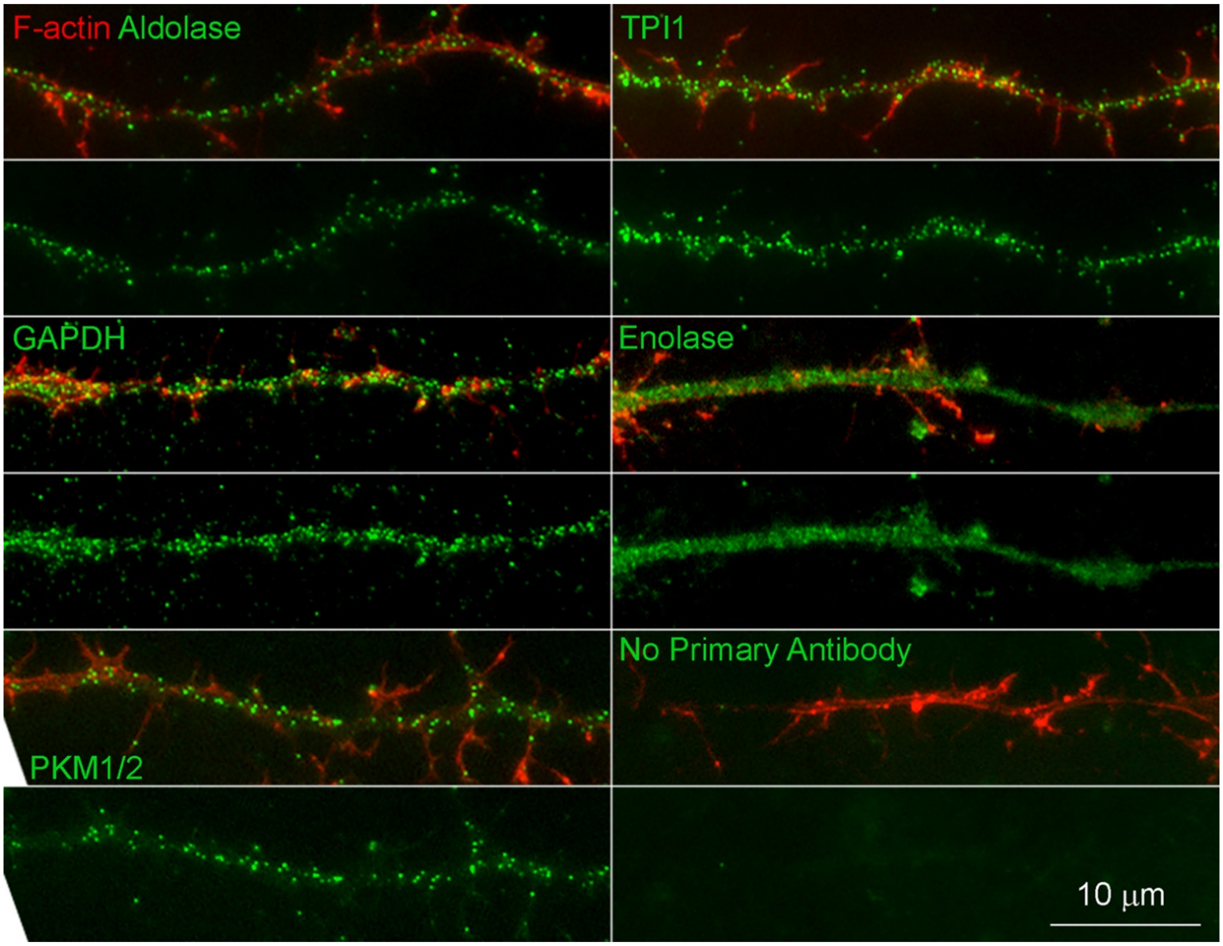

Supplemental Figure 3. Distribution of glycolytic enzymes along sensory axons. Images are taken from extant data sets from Ketschek et al (2021), wherein the distribution of enzymes in growth cones, but not axons, was specifically addressed. The no primary antibody panel show minimal background staining levels. Samples are counterstained with phalloidin to show actin filaments.

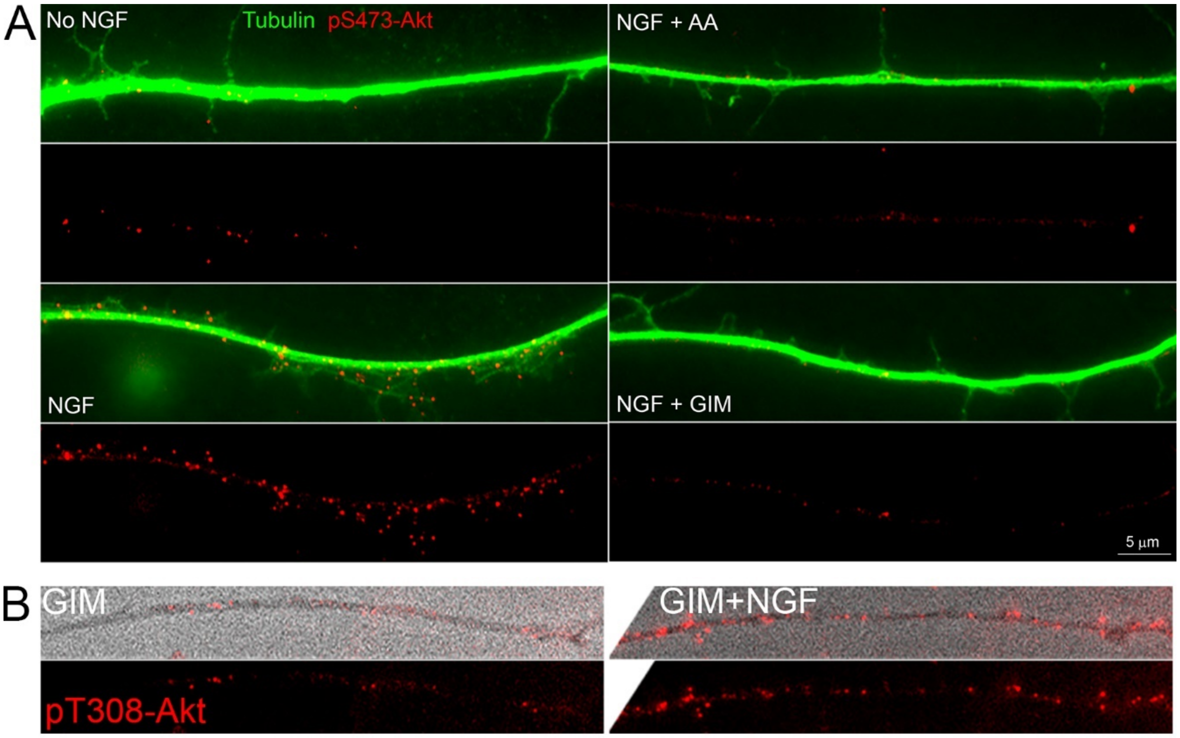

Supplemental Figure 4. Examples of the density of pT308-Akt and pS473-Akt puncta in conditions of inhibition of oxidative phosphorylation or glycolysis and acute treatment with NGF. **(A)** Examples of NGF treated axon with or without pretreatment with AA in support of Figure 4B. Pretreatment with AA blocks the NGF induced increase in the density of pS473-Akt puncta. Examples of NGF treated axons with GIM in support of Figure 4D. GIM pretreatment blocks the NGF induced increase in the density of pS473-Akt puncta. In this set of experiments samples were counter stained with tubulin antibodies to reveal axons and their morphology. **(B)** Examples of pT308-Akt staining in axons treated with NGF (no NGF) and pretreated with GIM and then NGF in support of Figure 4C. In the presence of GIM NGF treatment elevates the density of pT308-Akt puncta. Phase contrast overlay with pT308-Akt is shown. Scale bar in A also applies to B.

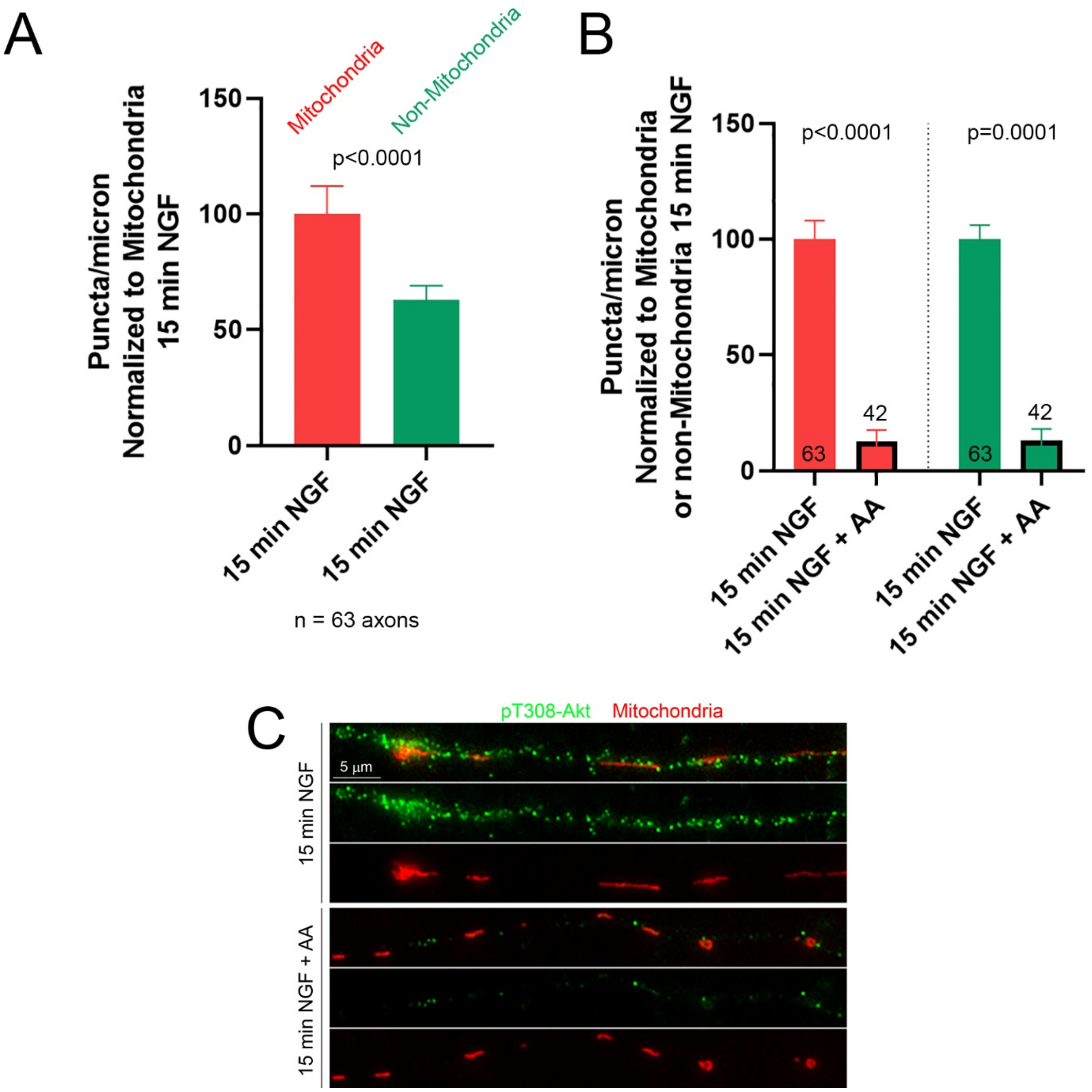

Supplemental Figure 5. Analysis of the effects of AA on NGF treatment (15 min) induced increases in pT308-Akt levels as a function of mitochondria positioning. **(A)** Extending the timeline from Figure B to 15 min of NGF treatment shows a consistent increase in the density of pT308-Akt levels in axon segments populated by mitochondria. **(B)** Analysis of the effects of AA pretreatment on the density of pT308-Akt puncta as a function of mitochondria position shows that AA equally suppresses density in axon segments populated by mitochondria and those not populated by mitochondria. **(C)** Examples of the density of pT308-Akt puncta in

relation to mitochondria in axons treated for 15 min with NGF with or without pretreatment with AA.

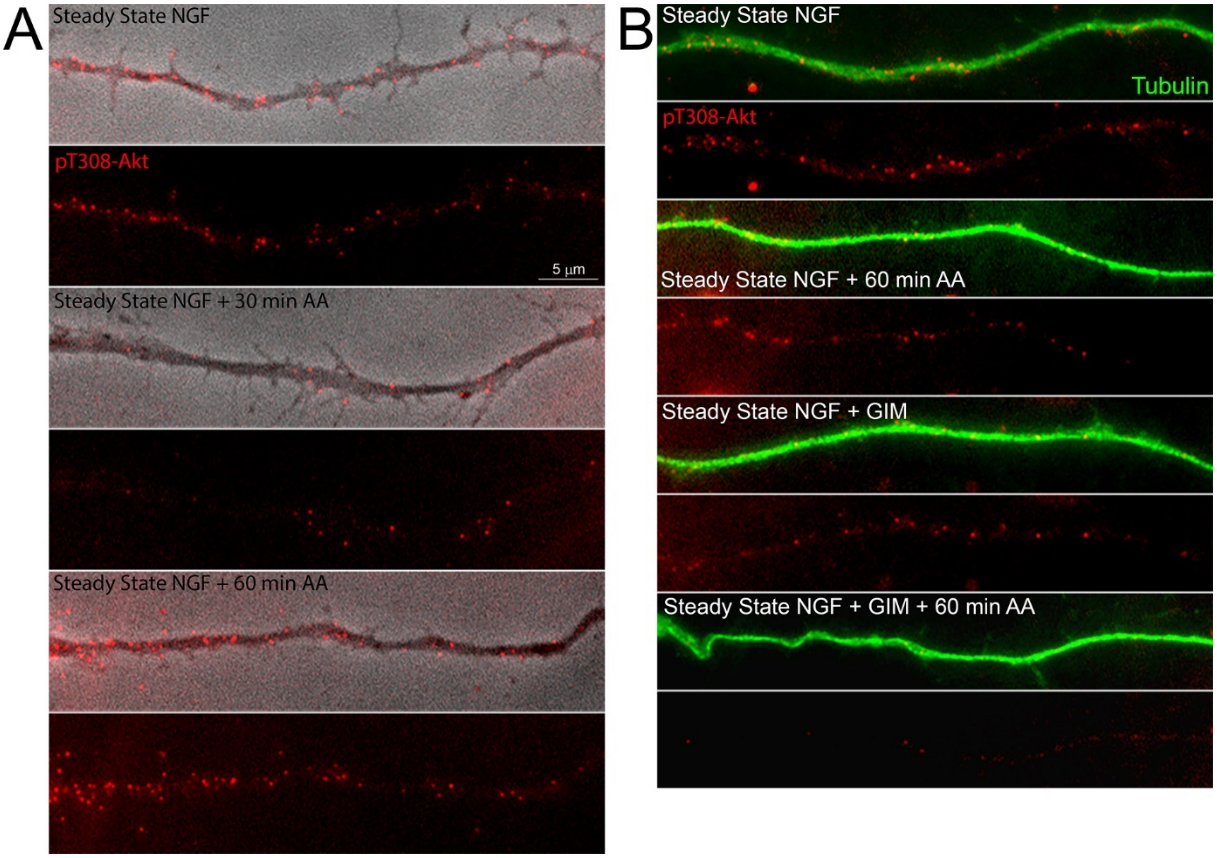

Supplemental Figure 6. Examples of the density of pT308-Akt puncta in conditions of inhibition of oxidative phosphorylation or glycolysis in steady state NGF. **(A)** Examples of puncta in support of the quantification shown in Figure 5A. A 30 minute treatment with AA suppresses the density of puncta, but the density recovers by 60 min of treatment. Phase contrast is shown to denote axons. **(B)** Examples of puncta in support of Figure 5C and D. As shown in Figure 5C and D, treatment with GIM decreases the density of puncta. The example shown here is from the experimental data set used to derived the quantification of Figure 5D. Treatment with GIM along with AA suppresses the restoration of puncta density at 60 minutes of treatment with AA (Figure 5D). The samples were counterstained with tubulin antibodies to reveal axons. The scale bar in (A) applies to B.

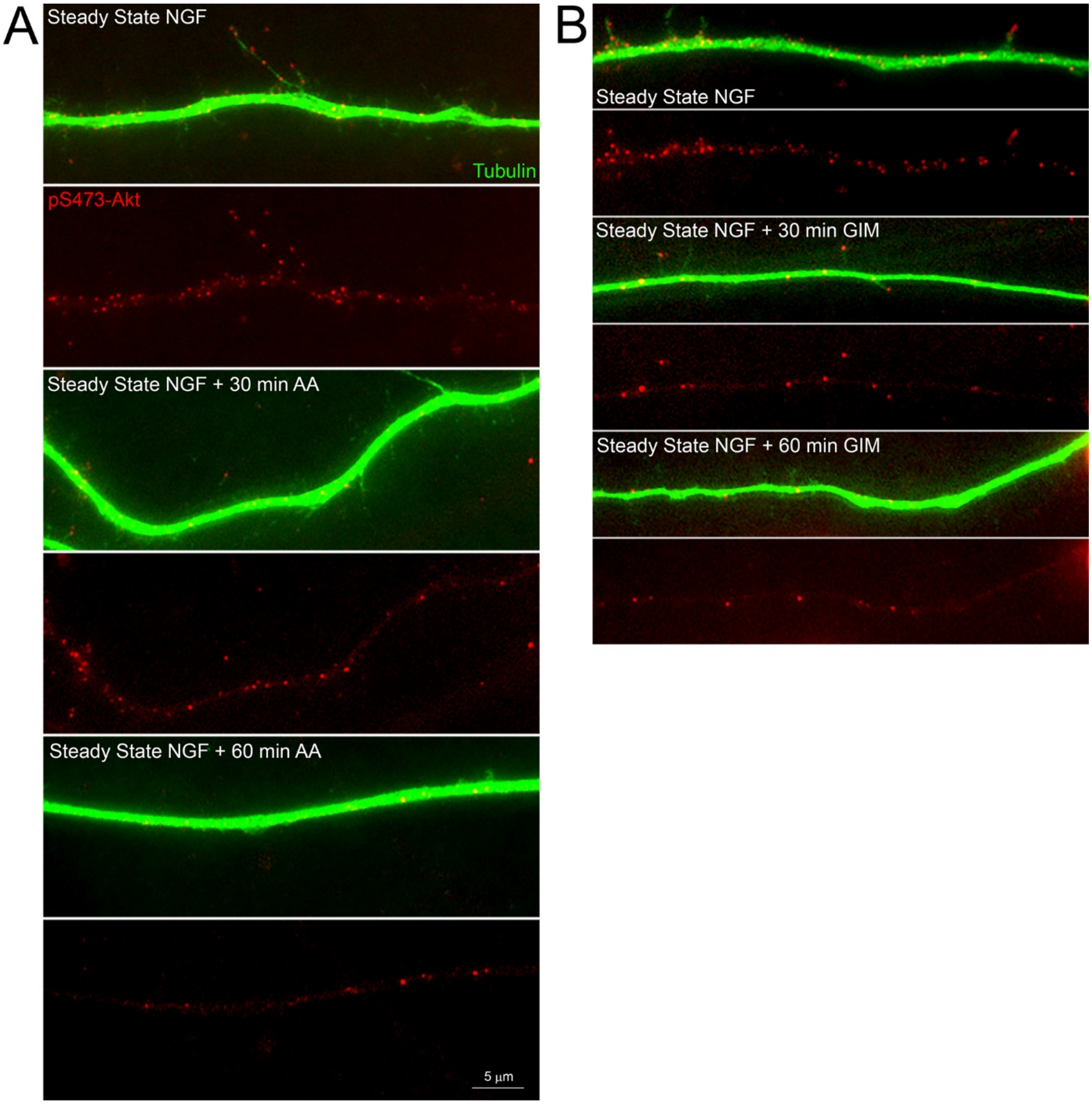

Supplemental Figure 7. Examples of the density of pS473-Akt puncta in conditions of inhibition of oxidative phosphorylation or glycolysis in steady state NGF. **(A)** Examples of puncta in support of the quantification in Figure 5E. A 60 min treatment with AA decreases puncta density, while a 30 min treatment has a non-statistically different effect on density. **(B)** Examples of puncta in support of the quantification in Figure 5F. A 30 or 60 min treatment with GIM suppresses the density of puncta. Scale bar and annotations in (A) apply to (B).

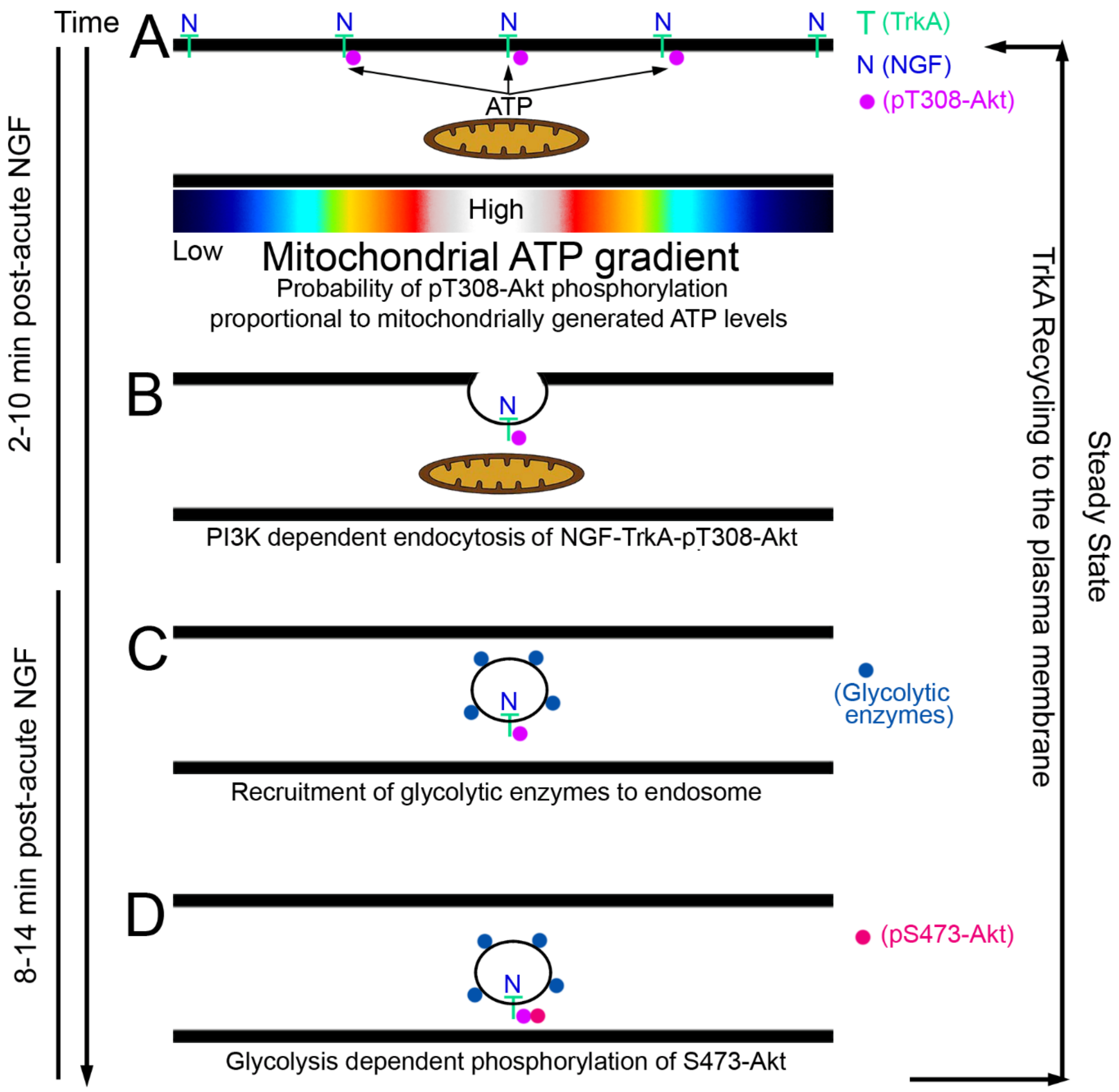

Supplemental Figure 8. Hypothetical mechanism of the sequential phosphorylation of Akt and the related bioenergetic requirements. **(A)** During acute NGF signaling, the time course shown on the left side of the schematic, NGF binds to the TrkA receptor and initiates the phosphorylation of Akt at T308. This occurs mostly in axon segments populated by mitochondria that provide the required ATP through oxidative phosphorylation. Due to the diffusion of ATP it

is expected that mitochondrially derived ATP levels will be highest at the mitochondrion and decline as a function of distance. The probability of the phosphorylation of Akt at T308 is proposed to be proportional to the mitochondria derived ATP level. This phosphorylation event occurs predominantly during the first 10 min of NGF signaling and is initially highest in the vicinity of mitochondria. **(B)** TrkA-NGF couplets are subsequently, or in concert with the phosphorylation at T308, endocytosed in a PI3K dependent manner (York et al., 2000; in main references) and the endosomes undergo cytoplasmic redistribution through transport thereby populating the non-mitochondria containing axon segments. Thus, inhibition of oxidative phosphorylation that suppresses T308 phosphorylation likely impacts the endocytotic event. **(C)** Endocytosed endosomes containing TrkA-NGF couplets recruit glycolytic enzymes on their surfaces. **(D)** The recruited glycolytic enzymes provide the ATP required for "on-board" phosphorylation of Akt at S473. Thus, failure to endocytose would prevent the phosphorylation of Akt at P473. Under steady state NGF signaling conditions, TrkA undergoes traffic to and from the plasma membrane and the cycle of phosphorylation (A-D) repeats as the TrkA recycles (right side of schematic).
